# VALIDATION OF HEART RATE MEASUREMENTS FROM THE POLAR VERITY SENSE DURING THE SUBMAXIMAL EKBLOM-BAK TEST IN ADULT INDIVIDUALS WITH OBESITY

**DOI:** 10.2340/jrm.v58.44975

**Published:** 2026-09-07

**Authors:** Grete Rudi ONSTAD, Fredrik Klæboe LOHNE, Marius Steiro FIMLAND

**Affiliations:** 1 Department of Neuromedicine and Movement Science, Faculty of Medicine and Health Sciences, NTNU Norwegian University of Science and Technology, Trondheim, Norway; 2 National Research Centre for the Working Environment, Copenhagen, Denmark; 3 Unicare Helsefort Rehabilitation Centre, Rissa, Norway

**Keywords:** obesity, heart rate, photoplethysmogram, Polar Verity Sense, Polar H10, Ekblom-Bak cycle test, validation, feasibility

## Abstract

**Objective:**

To assess the validity of Polar Verity Sense, an arm-worn heart rate (HR) monitor, for measuring HR in individuals with obesity during the submaximal ergometer cycle Ekblom-Bak test.

**Subjects/Patients:**

Adults in an inpatient rehabilitation programme for individuals with obesity.

**Methods:**

Participants completed the Ekblom-Bak test wearing the Polar H10 chest strap and 2 Verity Sense devices on the forearm and upper arm. Statistical analyses included intraclass correlation coefficient (ICC), Lin’s concordance correlation coefficient (CCC), Bland–Altman analyses with 95% limits of agreement (LoA), and mean absolute percentage error (MAPE). Feasibility was assessed with a post-test questionnaire.

**Results:**

49 participants were included in the analyses of estimated maximal oxygen consumption (VO_2_max), while 48 were included in analyses of HR data. ICC and CCC values ranged from 0.998–0.999 between the H10 chest strap and Verity Sense measures. Small differences were observed in Bland–Altman plots. MAPE ranged from 0.46% to 0.51%. Feasibility responses from the questionnaire showed that participants preferred the Verity Sense to the H10.

**Conclusion:**

Polar Verity Sense accurately measures HR and estimates VO_2_max in adults with obesity during the Ekblom-Bak test. Individuals with obesity may freely opt for either device without sacrificing accuracy during steady-state activities at low and moderate intensities. This choice may eliminate a barrier in assessing their cardiorespiratory fitness.

## LAY ABSTRACT

This study tested whether the Polar Verity Sense, a heart rate monitor worn on the arm, can accurately measure heart rate in people with obesity during the Ekblom-Bak cycle test, often used in rehabilitation. Traditionally, heart rate is measured using a chest strap, which some people may find uncomfortable. In this study, participants wore both the standard chest strap (Polar H10) and the Polar Verity Sense on the forearm and upper arm. The results showed that the Verity Sense measured heart rate almost identically to the chest strap. It also gave reliable estimates of fitness level (VO_2_max). In addition, most participants reported preferring the arm-worn device over the chest strap. These findings suggest that the Polar Verity Sense is an accurate, comfortable, and user-friendly alternative to chest strap monitors for people with obesity, making it easier to complete fitness tests and control exercise intensity in exercise rehabilitation programmes.

Obesity is a major global health challenge that increases risk of cardiovascular diseases, diabetes, and several other diseases (1). Cardiorespiratory fitness (CRF) is closely linked to cardiovascular health, making its accurate assessment essential for disease prevention and management (2). Developing practical and reliable methods for evaluating CRF is therefore crucial to improve cardiovascular health outcomes in individuals with obesity.

CRF assessments help evaluate clinical risk and optimize treatment strategies. Regularly measuring CRF provides valuable insights into treatment effectiveness, including guidance on engaging in physical activity (3). Traditionally, CRF is assessed by measuring maximal oxygen consumption (VO_2_max) during a cardiopulmonary exercise test (CPET). However, CPET is time-consuming, expensive, and requires specialized equipment and expertise. As assessment of VO_2_max during CPET typically requires maximal or near-maximal effort, attaining the required intensity may be challenging for some individuals with obesity, supporting the use of submaximal alternatives (4). Consequently, submaximal performance tests have been developed, improving access to CRF assessment in rehabilitation settings (3). For individuals with obesity, cycle ergometry may be a preferred CRF assessment method (5). The Ekblom-Bak Cycle Ergometer Test is a submaximal exercise protocol estimating VO_2_max (4). It involves cycling at low and higher individualized work rates, with heart rate (HR) monitored during the final minute of each workload. The VO_2_max estimate is derived from the HR response to exercise; therefore, accurate HR measurement is essential for valid estimation. Evaluating alternative HR monitors in this setting is thus clinically relevant.

The Polar H10 chest strap, commonly used for HR monitoring, has shown high agreement with the electrocardiograph (6). However, challenges with chest strap monitors include discomfort, skin irritation, reliance on assistance for placement, and connectivity issues (7, 8). These challenges, particularly relevant for obese individuals with excessive adipose tissue, have made user-friendly alternatives like arm-worn HR monitors interesting. Arm-worn HR sensors can offer greater practicality than chest-worn monitors by reducing sweat and skin irritation, which can cause discomfort (7, 8). Additionally, whereas chest straps require the user to expose their chest, the arm-worn HR sensor can be placed without undressing. Given these practical advantages, usability factors such as comfort, convenience, and ease of independent placement may influence device acceptance and feasibility in clinical and rehabilitation settings. Arm-worn HR sensors, such as the Polar OH1 and Polar Verity Sense, use non-invasive photoplethysmography (PPG) technology (9, 10). Studies affirm their validity during light and moderate exercises (7, 8, 11–14), though findings are largely limited to young, normal-weight subjects, limiting the generalizability to individuals with obesity. However, obesity-related characteristics such as greater subcutaneous adipose tissue and larger limb circumference, together with factors known to affect PPG measurements such as peripheral perfusion and motion artefact, may influence signal quality and HR measurement accuracy (7, 8, 12). Sensor performance may also vary according to placement site, as tissue composition and device contact can differ between forearm and upper arm (12, 15). Together, these factors underscore the importance of evaluating the Polar Verity Sense in individuals with obesity.

This study investigates the validity and usability of the Polar Verity Sense, worn on the forearm and upper arm, compared with the Polar H10 chest strap among individuals with obesity. Specifically, we compare steady-state HR results during the low and higher individualized work rates of the Ekblom-Bak test, VO_2_max estimates from the Ekblom-Bak test, as well as user preferences and ease of device placement.

## METHODS

### Participants

Participants were recruited from an inpatient rehabilitation programme for individuals with obesity. This study used a convenience sample approach, including every patient willing to participate between September and December 2023. The inclusion criteria for the rehabilitation programme were a BMI ≥ 35 with obesity related comorbidities or a BMI ≥ 40 regardless of comorbidity status (16). As the rehabilitation programme consisted of 4 stays during a 1-year period, BMI varied at the time of testing, ranging from 29.8 to > 40 kg/m^2^. At the time of testing, 1 participant was classified as overweight (BMI 29.8), whereas the remaining participants met the criteria for obesity: 15 had obesity class 1 (BMI 30-34.9), 14 had obesity class 2 (BMI 25-29.9), and 20 had obesity class 3 (BMI > 40). Characteristics of the study sample are presented in Table I. No formal *a priori* sample size calculation was conducted for the validation analysis, as the sample size was decided by the number of available participants from the rehabilitation programme.

**Table I:** Characteristics of the study sample

Demographics	All participants (*n* = 49)		Men (*n* = 10)		Women (*n* = 39)	

	Mean (SD)	Range	Mean (SD)	Range	Mean (SD)	Range
Age (years)	48.0 (11.9)	23–69	48.0 (12.9)	23–60	48.0 (11.9)	23–69
Height (cm)	170.1 (10.2)	151.0–206.0	184.3 (9.1)	173.0–206.0	166.5 (6.7)	151.0–182.7
Weight (kg)	112.6 (21.9)	79.3–195.2	134.0 (27.3)	103.0–195.2	107.1 (16.7)	79.3–141.4
BMI	38.8 (5.7)	29.8–54.2	39.2 (6.0)	29.8–48.7	38.7 (5.7)	30.0–54.2

SD: standard deviation; BMI: body mass index.

Patients received both oral and written information concerning the project during a plenary meeting at the rehabilitation centre. The examiner restated the information to each participant; all were required to provide written consent prior to conducting the Ekblom-Bak test and a questionnaire. The study adhered to the principles of the Declaration of Helsinki and received approval from the Norwegian Centre for Research Data (sikt.no; project ID: 522353).

### Ekblom-Bak test

The Ekblom-Bak tests were performed on a Monark model 928E ergometer cycle with electric brakes (Vansbro, Sweden), while participants were equipped with three Polar HR monitors (Kempele, Finland). Before commencing the test, participants were provided with information regarding the Ekblom-Bak test and the Borg’s Rating of Perceived Exertion scale (Borg RPE; 6–20 scale). Participants were instructed to pedal with a cadence of 60 revolutions per minute in accordance with the standardized Ekblom-Bak protocol. The test lasted ~8 min, with the initial 4 min performed at a fixed work rate of 30 W. This was directly followed by 4 min at a higher individualized work rate customized by the researcher based on clinical judgement. Workload selection was based on the participants’ sex, body size, self-reported training level, and prior experience with cycle ergometry, aiming to achieve a Borg RPE of approximately 14 by the last minute. After the first minute at the higher work rate, participants assessed their current Borg RPE. If their reported score was below 12, the work rate was increased, and participants were asked to reassess their Borg RPE after 1 min at the new work rate. This increase in work rate was implemented a maximum of 3 times. If the work rate was adjusted, the 4-min period at the higher work rate was restarted to ensure steady-state conditions were achieved at the final workload. Finally, participants were asked to assess their Borg RPE after 4 min on the higher work rate to ensure it corresponded to a Borg RPE of approximately 14. In accordance with the rehabilitation centre’s protocol, participants who had previously undergone the test adhered to the same work rate and duration as in their prior assessments. The procedures used in the present study were performed in accordance with the standardized Ekblom-Bak protocol described on the Swedish School of Sport and Health Sciences’ website (17).

The equations utilized in the Ekblom-Bak test incorporate the difference in HR between the 2 work rates, relative to the increase in power output (PO), to calculate VO_2_max. Independent variables for these equations include sex, age, and weight, alongside the average HR recorded during both work rates. According to the original protocol, HR is recorded at 15-s intervals during the final minute of each work rate and averaged to calculate the mean HR for each work rate. In the present study, mean HR for each work rate was calculated as the average of all recorded HR values obtained during the final minute. Participant’s weight was registered to the nearest 0.1 kg and used to estimate their VO_2_max (ml/kg/min). To calculate the estimated VO_2_max for the Ekblom-Bak test, an Excel (Microsoft Corp, Remond, WA, USA) sheet provided by the Swedish School of Sport and Health Sciences was used (17). This sheet included the formula (4) for the latest update to the sex-specific equations. For women, the equation used was: VO_2_max = 1.84390 − 0.00673 (age) − 0.62578 (ΔHR/ΔPO) + 0.00175 (ΔPO) − 0.00471 (HR at standard work rate), and for men the equation was: VO_2_max = 2.04900 − 0.00858 (age) − 0.90742 (ΔHR/ΔPO) + 0.00178 (ΔPO) − 0.00290 (HR at standard work rate) (4).

### Devices

Prior studies have affirmed Polar H10’s validity for HR measurement (6). It is an ECG-based HR sensor secured around the chest using the accompanying Polar Pro Strap, with the electrode area positioned over the sternum at the xiphoid process. HR is derived from electrical cardiac signals detected by the electrodes embedded in the strap.

Polar Verity Sense employs PPG technology by emitting light onto the skin to detect changes in blood volume within the microvascular tissue beneath the skin surface (9, 10). These alterations in blood volume influence the quantity of light reflected to the sensors, thereby facilitating the determination of HR. The device is attached to an armband worn on either the forearm or upper arm. In the current study, 2 Verity Sense devices were employed, 1 placed on the forearm and 1 on the upper arm, both on the non-dominant arm. To reduce potential bias, device placement was alternated systematically between participants, with the devices switching between forearm and upper arm placement for each consecutive participant. HR data collected from the devices were transferred to the PerformTek app version 2.0.1 (Valencell Inc, Raleigh, NC, USA) via an iPhone. The application continuously collected and exported processed HR values at 1-s intervals from each device rather than raw PPG data, allowing temporal alignment of the recordings for subsequent analyses without additional correction for temporal lag between devices. For analysis, all recorded HR values during the final minute of both work rates were averaged to calculate mean HR. Exported data were manually reviewed for missing values and synchronization errors prior to analysis.

Furthermore, the investigator provided instructions to participants on how to correctly position and remove the HR devices. The investigator recorded whether each participant was able to independently place the devices prior to the test and remove them after the test. In cases where a device was incorrectly placed, the investigator made the necessary adjustments to ensure proper placement and measurements for the Ekblom-Bak test. Before initiating data collection, the investigator also verified that all devices were transmitting a signal to ensure they were recording throughout the test.

### Questionnaire

Following completion of the test, participants answered questions verbally from a questionnaire assessing their perspectives on the HR devices. The questionnaire consisted of 7 questions covering prior use of HR monitoring, comfort with using a chest belt in a gym setting, perceived discomfort with the devices, and device preference. Participants were also asked to rank the 3 devices from most to least preferred, based on their overall experience, including comfort and usability. The preferences are analysed and presented descriptively. Two questions were open-ended, allowing participants to elaborate on their experiences with the devices and the test protocol; these responses were analysed using a simple thematic approach. The questionnaire was developed for this study.

### Statistical analyses

All statistical analyses were conducted using SPSS statistical software version 29.0.1.0. (IBM Corp, Armonk, NY, USA). Descriptive statistics, including mean and standard deviation (SD), were calculated for descriptive data including age, height, weight, and BMI.

The validity of the Polar Verity Sense was assessed separately for the forearm and upper arm placements by comparison with the Polar H10 for the average HR during the final minute of each workload, and the estimated VO_2_max. HR data were visually inspected for normal distribution using histograms and quantile–quantile plots, and were found to be normally distributed, presented as mean±SD.

Bland–Altman analyses were performed to determine the agreement between the H10 and the Verity Sense devices, identify potential systematic differences, and identify data points with lower agreement (18, 19). A 95% limit of agreement (LoA) was calculated as mean bias±1.96 x SD of the differences.

Mean absolute percentage error (MAPE) and Lin’s concordance correlation coefficient (CCC) were used to assess criterion validity. MAPEs of 0–5% were within acceptable limits, consistent with previous studies (20, 21). CCC values were interpreted according to previously proposed agreement thresholds, with values ≥ 0.90 considered acceptable (22).

Agreement between Verity Sense and H10 was determined using an intraclass correlation coefficient (ICC) (single measure, two-way mixed, absolute agreement). Adhering to recommended classifications (23, 24), values were classified as poor (< 0.50), moderate (0.50–0.75), good (0.75–0.90), and excellent (> 0.90) based on the 95% confidence interval of the ICC. The correlation was considered significant if the *p*-value was < 0.05.

## RESULTS

**Fig. 1:**
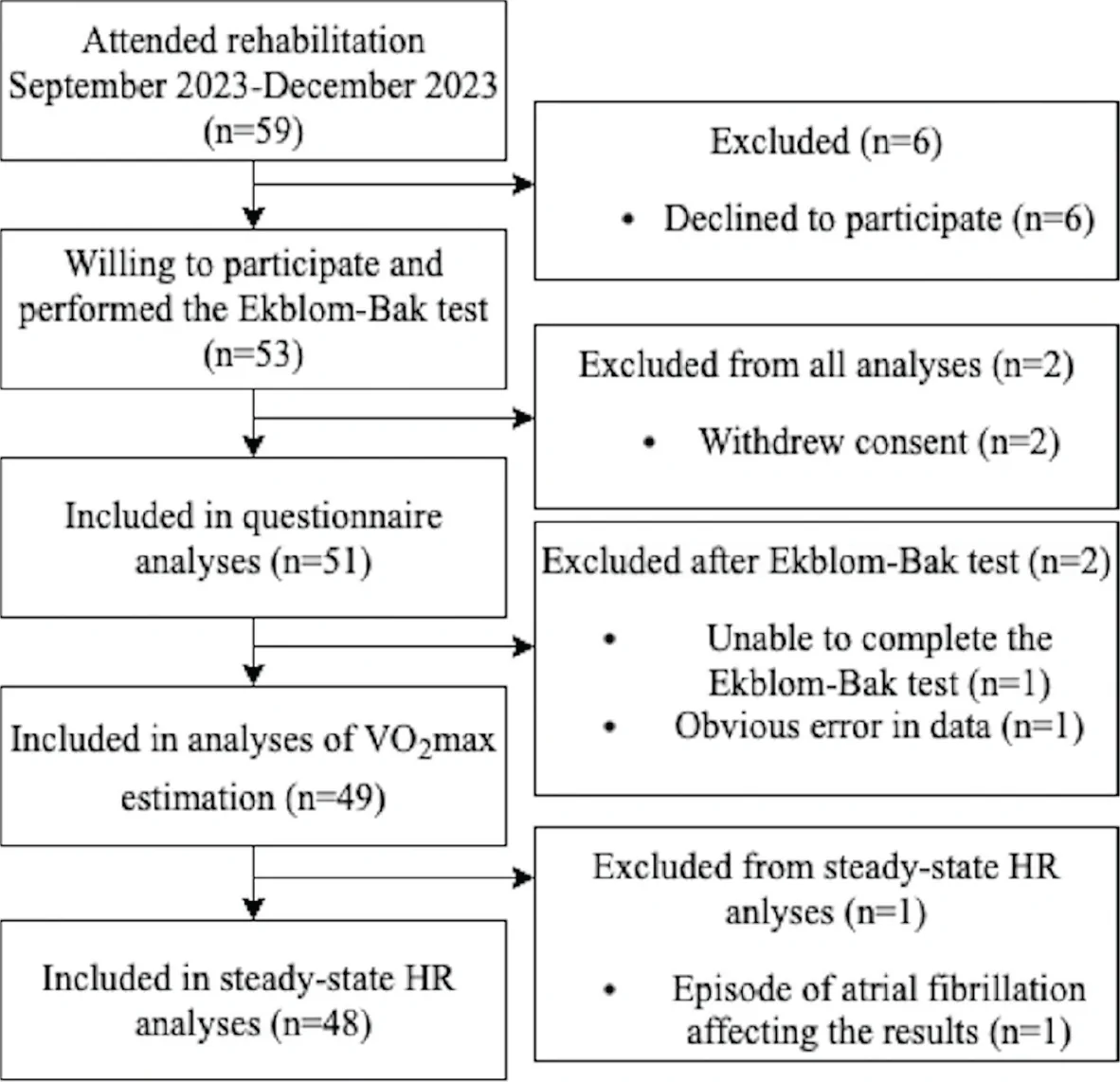
Flowchart describing the recruitment and exclusion process.

The recruitment and exclusion process are presented in Fig. 1. A total of 59 individuals attended the rehabilitation programme during the recruitment period, of whom 53 were willing to participate and performed the Ekblom-Bak test. Two participants later withdrew consent, resulting in 51 participants being included in the feasibility analyses. Two participants were excluded from the HR and the VO_2_max analyses, as 1 participant was unable to complete the Ekblom-Bak test and 1 participant had an obvious error in the criterion device recording. The recording error affected only the criterion device (Polar H10), whereas recording from the Verity Sense devices was successfully obtained. Additionally, 1 participant was excluded from the steady-state HR analyses due to an episode of atrial fibrillation affecting the HR recording. Consequently, the estimated VO_2_max analyses included 49 participants, whereas the steady-state HR analyses included 48 participants. For the steady-state HR analyses, 2 averaged HR values per participant (1 from each workload) were included in the analyses. Each averaged HR was calculated from 60 second-by-second HR recordings obtained during the final minute of each workload.

### Validity

One participant was excluded from the validity analyses due to connectivity issues with the criterion device (Polar H10). Although HR data were successfully recorded using the Verity Sense, the absence of corresponding data from the H10 prevented inclusion in the validity analyses.

Table II presents descriptive statistics and validation metrics for estimated VO_2_max and measured HR. ICC and CCC values ranged from 0.998 to 0.999, while corresponding MAPE values ranged from 0.46% to 0.51%.

**Table II:** Descriptive statistics and validation metrics of estimated VO_2_max and measured heart rate

Factor	Chest	Forearm	Upper arm
Estimated VO_2_max, ml/kg/min, mean (SD)	26.87 (4.39)	26.90 (4.43)	26.89 (4.42)
Mean bias (95% CI), ml/kg/min		–0.025 (-0.084 to 0.034)	–0.016 (–0.077 to 0.044)
LoA, ml/kg/min		–0.422 to 0.373	–0.429 to 0.396
MAPE (%)		0.51	0.49
Intraclass correlation coefficient (95% CI)		0.999 (0.998 to 0.999)	0.999 (0.998 to 0.999)
Lin’s concordance correlation coefficient		0.999	0.999
Low work rate, bpm, mean (SD)	98.49 (13.37)	98.27 (13.25)	98.27 (13.22)
Mean bias (95% CI), bpm		0.22 (0.02 to 0.43)	0.22 (-0.02 to 0.46)
LoA, bpm		-1.15 to 1.60	-1.40 to 1.84
MAPE (%)		0.47	0.51
Intraclass correlation coefficient (95% CI)		0.999 (0.997 to 0.999)	0.998 (0.996 to 0.999)
Lin’s concordance correlation coefficient		0.999	0.998
Higher work rate, bpm, mean (SD)	126.77 (14.41)	126.50 (14.55)	126.53 (14.55)
Mean bias (95% CI), bpm		0.27 (0.04 to 0.50)	0.24 (0.03 to 0.45)
LoA, bpm		-1.28 to 1.81	-1.18 to 1.66
MAPE (%)		0.51	0.46
Intraclass correlation coefficient (95% CI)		0.998 (0.997 to 0.999)	0.999 (0.997 to 0.999)
Lin’s concordance correlation coefficient		0.998	0.999

VO_2_max: maximal oxygen consumption; SD: standard deviation; CI: confidecne interval; LoA: limits of agreement; MAPE: mean absolute percentage error. MAPE and Lin’s concordance correlation coefficient were used as measures of validity, whereas intraclass correlation coefficient was used as a measure of agreement.

**Fig. 2:**
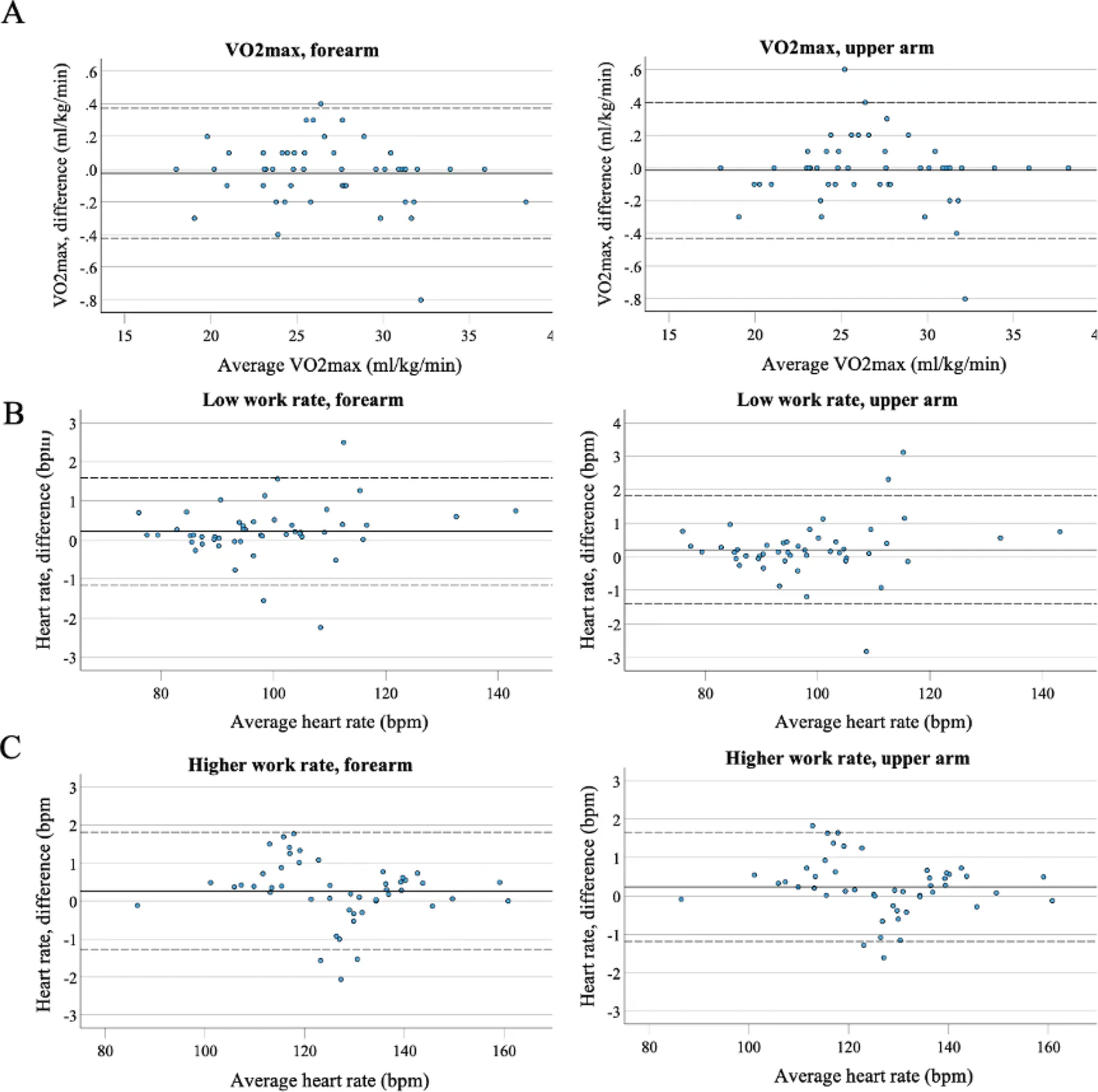
Bland–Altman plots with bias (bold line) and 95% limits of agreement (dotted lines) comparing Polar H10 and Polar Verity Sense. Panel A shows the results for the VO_2_max estimation analyses, panel B shows the results for the low work rate HR analyses, and panel C shows the results for the higher work rate HR analyses. The x-axis represents the mean of the Polar H10 and the Polar Verity Sense measurements for each participant. HR values were calculated as the average HR during the final minute of each workload.

The Bland–Altman plots (Fig. 2) illustrate the mean bias and 95% LoA between the Polar Verity Sense and the Polar H10 across all testing conditions. VO_2_max estimates (A) showed mean differences of –0.025 ml/kg/min for the forearm and –0.016 ml/kg/min for the upper arm placements, respectively, with corresponding 95% LoA of –0.442 to 0.373 and –0.429 to 0.396. At low work rate (B), HR measurements showed mean bias values of 0.22 bpm for both the forearm and upper arm placements, with corresponding LoA of –1.15 to 1.60 and –1.40 to 1.84, respectively. At higher work rate (C), forearm placement showed a mean bias of 0.27 bpm and 95% LoA of –1.28 to 1.81, whereas the upper arm placement showed a mean bias of 0.46 bpm and 95% LoA of –1.18 to 1.66.

### Usability

Difficulty with device placement was assessed by the investigator and defined as requiring assistance or adjustment to achieve correct device positioning. Using this definition, 5 of the 51 participants were unable to correctly place any of the devices, 26 participants successfully placed both arm-worn devices but required assistance or adjustments for the chest-worn device, and 20 participants correctly positioned all devices independently.

Among the 51 participants, 4 reported discomfort from the chest strap, 2 with the Verity Sense on the upper arm (due to tightness despite maximum strap length), and none with the forearm placement. The remaining 45 reported no discomfort with any device.

**Fig. 3:**
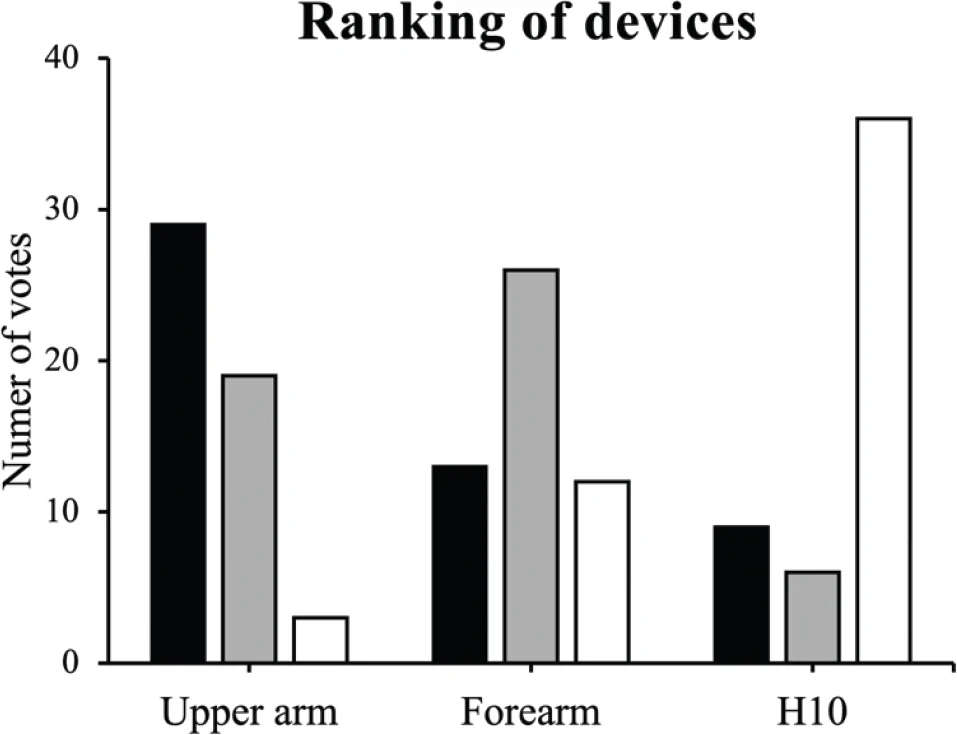
Ranking of the HR measurement devices based on participants’ overall experience, including comfort and usability. Black: first choice; grey: second choice; white: third choice. The figure illustrates the distribution of participant ranking across the 3 devices.

Participant preferences varied: 9 favoured the H10, 13 preferred the Verity Sense on the forearm, and 29 preferred the Verity Sense on the upper arm (Fig. 3). When asked about adjusting a chest strap in a gym setting – considering factors like limited privacy and the need to lift or remove clothing – 26 participants reported it as problematic, while 25 did not.

### Subgroup analyses

Supplementary subgroup analyses stratified by sex and BMI were conducted for HR at the low and higher work rates and estimated VO_2_max using agreement and validity metrics. No meaningful subgroup differences were observed. Detailed results are provided in Tables SI and SII.

## DISCUSSION

The main findings of this study were that heart rate measurements from the arm-worn Polar Verity Sense very closely matched those from the Polar H10 chest measurements during steady-state cycling at the low and higher work rates of the Ekblom-Bak test in individuals with obesity. Consequently, the estimated VO_2_max values from the Ekblom-Bak test were nearly identical. Furthermore, the Verity Sense was preferred by most participants, and easier to position correctly. To our knowledge, this is the first study comparing arm- and chest-worn heart rate monitors in individuals with obesity.

The current study demonstrates strong criterion-related validity of the Verity Sense compared with the H10, with findings indicating a high degree of agreement between the 2 devices. Previous studies of the Polar OH1, the precursor to the Verity Sense, examined HR monitoring in healthy young and adult individuals across a range of exercise intensities, including moderate to vigorous intensities (11) and light to vigorous intensities (7, 8). Although these studies generally reported small mean biases, their LoA were wider than those observed in the present study. In addition, a previous study of the Verity Sense during trail running demonstrated acceptable validity relative to a chest strap monitor, although performance varied depending on the exercise segment (12). In contrast, the current study, conducted exclusively in individuals with obesity, showed consistently low mean bias and narrow LoA at both low and higher work rates, underscoring the validity of the Verity Sense for HR monitoring during submaximal exercise conditions. The combination of low mean bias, narrow LoA, and excellent agreement coefficients further supports the strong agreement observed between the devices when examining HR at low and higher work rates. These findings are also consistent with those reported by Schweizer and Gilgen-Ammann (14), who demonstrated excellent agreement between the Polar Verity Sense and the Polar H10 across several exercise modalities, including cycle ergometer exercise. However, direct comparisons between studies should be interpreted cautiously because they involve different PPG devices, study populations, exercise protocols, data processing approaches, and statistical methods. Together, these findings support the validity of the Verity Sense during submaximal cycle ergometer testing.

The higher agreement observed between the devices in the present study may, in part, be attributed to the choice of activity. Unlike previous studies that involved activities with greater upper body movement, such as trail running (12), yoga (7), or combinations of treadmill and ergometer cycling (8, 11), the current study exclusively employed ergometer cycling. This mode of exercise involves minimal arm movement, which likely reduces the motion artefacts and signal disruption, thereby enhancing the stability and accuracy of the HR measurements.

The Bland–Altman analyses demonstrated small mean bias and relatively narrow LoA between the Polar Verity Sense and the Polar H10 across both HR and estimated VO_2_max measurements, supporting the agreement between the devices during the Ekblom-Bak test. Both placements demonstrated small deviations relative to the criterion device. Compared with previous findings by Schubert et al. (7), who reported HR underestimations of –6 and –7 bpm, the deviations observed in the current study were smaller, indicating closer agreement between the devices during steady-state cycling in individuals with obesity.

All MAPE values from the HR data fell within the predefined acceptable range of 0–5%, with values between 0.46% and 0.51%. These discrepancies were less pronounced than those reported by Navalta et al. (12) in their validation of the Verity Sense during trail running. Additionally, the MAPE values obtained in this study align closely with those reported by Muggeridge et al. (11) in their evaluation of Polar OH1 in conditions involving low and moderate physical activities.

In addition to evaluating the validity of the Verity Sense for HR measurement, the present study also examined whether the HR measurements obtained from the device produced VO_2_max estimates comparable to those derived from the H10 during the Ekblom-Bak test in individuals with obesity in a clinical setting. Despite a thorough literature search, no prior studies were found with this aim. The close agreement observed for HR measurements between the Verity Sense and the H10 was likewise reflected in the corresponding VO_2_max estimates. However, because the VO_2_max estimates were calculated from HR measurements from the Ekblom-Bak test, this agreement does not represent an independent validation of VO_2_max but largely reflects the close agreement in HR between the devices. Nevertheless, the analysis remains clinically relevant, as it demonstrates that HR measurements obtained with the Verity Sense produce VO_2_max estimates comparable to those obtained with the Polar H10. The very low MAPE values, minor underestimations, and narrow LoA indicate that the differences between the devices are unlikely to be clinically meaningful. Thus, both forearm and upper-arm placement of the Verity Sense appear suitable for generating VO_2_max estimates during the Ekblom-Bak test.

Prior to conducting the Ekblom-Bak test, participants received instructions to position the HR devices independently in accordance with the investigator’s guidance. Among the 51 participants, 5 individuals were unable to position any of the devices correctly, 26 accurately placed the arm-worn devices, and 20 participants successfully positioned all devices. Although previous studies (7, 8) did not assess the feasibility of the devices through questionnaires, reduced comfort and increased sweat were reported with chest-worn devices. These findings align with the results of the current study. The questionnaire findings further suggest a clear participant preference for arm-worn HR monitoring compared with chest-worn monitoring.

Upon investigating the questionnaire responses, several factors appeared to contribute to the prevalent preference for the Verity Sense over the H10 among most participants. Half of the participants expressed discomfort with the possibility of lifting their shirt to place and adjust the chest belt in a crowded gym setting, highlighting practical advantages associated with an arm-worn HR monitor. Moreover, the less intrusive placement of the arm-worn device may enhance the comfort of using HR monitors, where the need for participants to lift their shirts is eliminated, and self-placement of an arm-worn device is possible for most participants. This potential for self-placement was supported when participants attempted to position the devices, with the Verity Sense being the device that most participants managed to place correctly. As the questionnaire responses are analysed descriptively, these findings should be interpreted as participant-reported experiences rather than statistically analysed differences between devices.

However, while participants generally responded positively to the Verity Sense devices according to questionnaire data, 2 individuals reported discomfort with upper-arm placement due to tightness. This finding highlights a limitation of a “one size fits all” design, which may not accommodate variation in body size and shape across users. This issue was expected, given Polar’s restriction of the device to a single size. Considering that both forearm and upper arm placement demonstrated validity relative to the H10, individuals experiencing discomfort with upper arm placement may use the device on the forearm without compromising validity.

The present study adhered to the Ekblom-Bak test protocol, focusing on HR measurement at steady-state levels corresponding to low and higher work rates. Investigating the validity of the Verity Sense during intensity fluctuations would be beneficial, given the lack of studies addressing this aspect. Nonetheless, prior research (11) has assessed the validity of Polar OH1, finding it less accurate in measuring HR during high-intensity exercises. Moreover, a study (22) on PPG monitors reported poor validity during running with fluctuating intensity in natural settings. These findings have raised concerns as to whether similar issues may affect the Verity Sense (12). Consequently, further investigations are warranted to assess whether fluctuating intensity and exercise types impact the validity and reliability of the Verity Sense.

### Practical recommendations

Measurements obtained from both arm placements demonstrated high validity for HR and estimated VO_2_max, compared with those obtained with the H10 chest strap. Therefore, the Polar Verity Sense can confidently be used to monitor HR during steady-state cycling at low and higher work rates within the Ekblom-Bak protocol for individuals with obesity. Additionally, questionnaire results revealed that most participants preferred the arm-worn device over the chest strap, making it more favourable for both institutional and personal use.

### Strengths and limitations

A strength of this study is the placement of the arm-worn HR sensors. Navalta et al. (12) acknowledged that positioning the devices on each arm might impact the validity of the sensors, as variations in blood flow patterns between limbs could arise. However, in the present study, both arm-worn devices were positioned on the same arm, thereby alleviating this potential limitation. Furthermore, although prior studies have addressed participants’ perceived feasibility of using HR sensors, this study is the first to systematically assess user experience through a structured questionnaire.

Another strength is the sample size. While previous studies with similar aims often have smaller sample sizes, typically ranging from 15 to 40 participants (7, 8, 11, 12), the present study included 49 participants in the estimated VO_2_max analyses and 48 in the HR measurements. This aligns with recommended guidelines for the validation of consumer-grade HR monitor devices (25), thus strengthening the robustness of the findings. However, it should be noted that the validity analyses in the present study were based on 1 averaged HR value from each participant at each work rate. As a result, the number of paired comparisons was lower than the total number of recorded HR measurements, which should be considered when interpreting the findings. The study population also presents a significant strength. To date, no known research has specifically examined HR monitoring in individuals with obesity, a population at increased risk of cardiovascular health issues. As such, the findings offer valuable insight into the applicability and accuracy of wearable HR technology within this underserved group.

Although ECG is widely regarded as the gold standard for HR measurement, practical constraints at the rehabilitation facility prevented its use in the current study. Instead, the Polar H10 was used as the criterion device, as previous studies have demonstrated strong agreement between the H10 and ECG during exercise testing (6). Nevertheless, the use of the H10 instead of ECG may be considered a limitation, as ECG-based recording could have provided a more direct assessment of cardiac electrical activity. Additionally, participants who had previously completed the Ekblom-Bak test at the rehabilitation facility performed the test using the same workload as their prior assessment, in accordance with the facility’s protocol. Although this approach was intended to standardize repeated assessments, it may have reduced the individualization of workload selection, as previous assigned workloads were retained rather than reassessed before testing. This was reflected in 1 participant being unable to complete the test due to excessive workload.

### Conclusion

Among adults with obesity, the Polar Verity Sense accurately measures steady-state HR and enables VO_2_max estimation during the Ekblom-Bak test, with strong agreement observed relative to the Polar H10. Both forearm and upper-arm placements appear suitable for use during steady-state cycling at low and higher work rates. The arm-worn design was also preferred by the participants, supporting its practicality and comfort in this population and suggesting that it may reduce barriers to cardiorespiratory fitness assessment.

## Supplementary Material


