# Supplementary material for: VALIDATION OF HEART RATE MEASUREMENTS FROM THE POLAR VERITY SENSE DURING THE SUBMAXIMAL EKBLOM-BAK TEST IN ADULT INDIVIDUALS WITH OBESITY
Source: J Rehabil Med. 2026 Sep 7;58:44975. doi: 10.2340/jrm.v58.44975 (PMC13559670; doi:10.2340/jrm.v58.44975)
Supplement: Supplementary file 1 [file JRM-58-44975-s1.pdf]

Supplemental material

Sensitivity analysis

Subgroup analyses further indicated that the Verity Sense provides valid HR measurements across both sexes and all three classes of obesity, irrespective of its placement on the upper arm or forearm. This suggests that either placement can be used interchangeably in clinical and practical settings, as they offer equally reliable measurements irrespective of sex or BMI classification.

Subgroup analyses – Sex

Supplementary Table I: Descriptive statistics and validation metrics of estimated VO_2max and measured heart rate stratified by sex.

	**Male**			**Female**		
	**Chest**	**Forearm**	**Upper arm**	**Chest**	**Forearm**	**Upper arm**
**VO_2max**						
Mean (SD) *	29.58 (4.13)	29.68 (4.22)	29.65 (4.19)	26.18 (4.23)	26.18 (4.25)	26.18 (4.24)
Mean bias (95% CI) *		-0.10 (-0.30 to 0.09)	-0.07 (-0.27 to 0.13)		-0.005 (-0.06 to 0.05)	-0.003 (-0.07 to 0.06)
LoA *		-0.639 to 0.439	-0.609 to 0.469		-0.356 to 0.346	-0.380 to 0.375
MAPE (%)		0.51	0.42		0.51	0.51
ICC (95% CI)		0.998 (0.992 to 0.999)	0.998 (0.992 to 0.999)		0.999 (0.998 to 1.000)	0.999 0.998 to 0.999)
CCC		0.998	0.998		0.999	0.999
**Low work rate**						
Mean (SD) **	89.99 (9.82)	89.66 (9.76)	89.66 (9.88)	100.45 (13.41)	100.25 (13.25)	100.26 (13.19)
Mean bias (95% CI) **		0.34 (-0.15 to 0.83)	0.34 (-0.12 to 0.80)		0.20 (-0.04 to 0.43)	0.19 (-0.09 to 0.48)
LoA **		-0.90 to 1.58	-0.84 to 1.51		-1.22 to 1.61	-1.53 to 1.91
MAPE (%)		0.56	0.60		0.45	0.49

ICC (95% CI)		0.998 (0.989 to 0.999)	0.998 (0.989 to 1.000)		0.998 (0.997 to 0.999)	0.998 (0.996 to 0.999)
CCC		0.997	0.998		0.998	0.998
**Higher work rate**						
Mean (SD) **	122.74 (20.82)	122.41 (20.88)	122.43 (20.94)	127.70 (12.68)	127.45 (12.86)	127.48 (12.83)
Mean bias (95% CI) **		0.32 (-0.10 to 0.75)	0.31 (-0.23 to 0.85)		0.25 (-0.02 to 0.53)	0.22 (-0.02 to 0.46)
LoA **		-0.76 to 1.40	-1.07 to 1.68		-1.39 to 1.90	-1.23 to 1.68
MAPE (%)		0.38	0.44		0.54	0.47
ICC (95% CI)		1.000 (0.998 to 1.000)	0.999 (0.997 to 1.000)		0.998 (0.996 to 0.999)	0.998 (0.996 to 0.999)
CCC		1.000	0.999		0.998	0.998

VO_2max: maximal oxygen consumption; SD: standard deviation, LoA: limits of agreement; MAPE: mean absolute percentage error; ICC: intraclass correlation coefficient; Pearson: Pearson moment correlation coefficient, CCC: Lin´s concordance correlation coefficient. MAPE and CCC were used as measures of validity, whereas ICC was used as a measure of reliability.

*ml/kg/min; **bpm

Subgroup analysis – BMI

Supplementary Table II: Descriptive statistics and validation metrics of estimated VO_2max and measured heart rate stratified by BMI classification.

	Class 1			Class 2			Class 3		
	**Chest**	**Forearm**	**Upper arm**	**Chest**	**Forearm**	**Upper arm**	**Chest**	**Forearm**	**Upper arm**
**VO_2max**									
Mean (SD) *	31 (4)	31 (4)	31 (4)	27 (4)	27 (4)	27 (4)	24 (4)	24 (4)	24 (4)
Mean bias (95% CI) *		-0.03 (-0.13 to 0.06)	-0.03 (-0.12 to 0.06)		0.03 (-0.06 to 0.12)	0.05 (-0.07 to 0.18)		-0.05 (-0.17 to 0.07)	-0.08 (-0.20 to 0.05)
LoA *		-0.37 to 0.32	-0.37 to 0.31		-0.27 to 0.33	-0.36 to 0.47		-0.31 to 0.45	-0.59 to 0.44
MAPE (%)		0.44	0.41		0.42	0.51		0.64	0.68
ICC (95% CI)		0.999 (0.997 to 1.000)	0.999 (0.997 to 1.000)		0.999 (0.997 to 1.000)	0.998 (0.994 to 0.999)		0.998 (0.994 to 0.999)	0.997 (0.993 to 0.999)
CCC		0.999	0.999		0.999	0.998		0.998	0.997
**Low work rate**									
Mean (SD) **	94 (9)	94 (9)	94 (9)	100 (17)	100 (17)	100 (17)	101 (13)	100 (13)	101 (13)
Mean bias (95% CI) **		0.19 (-0.33 to 0.72)	0.16 (-0.28 to 0.61)		0.37 (0.06 to 0.67)	0.48 (-0.71 to 1.03)		0.16 (-0.16 to 0.48)	0.09 (-0.30 to 0.47)
LoA **		-1.53 to 1.91	-1.29 to 1.64		-1.31 to 1.34	-1.31 to 2.27		-1.19 to 1.50	-1.54 to 1.71
MAPE (%)		0.45	0.39		0.48	0.59		0.48	0.54
ICC (95% CI)		0.993 (0.980 to 0.998)	0.995 (0.986 to 0.998)		0.999 (0.997 to 1.000)	0.998 (0.994 to 0.999)		0.999 (0.997 to 0.999)	0.998 (0.995 to 0,999)
CCC		0.993	0.995		0.999	0.998		0.999	0.998

**Higher work rate**									
Mean (SD) **	128 (16)	128 (16)	128 (16)	128 (13)	128 (13)	128 (13)	125 (15)	125 (15)	125 (15)
Mean bias (95% CI)**		0.31 (-0.14 to 0.74)	0.32 (-0.12 to 0.73)		0.26 (-0.01 to 0.53)	0.21 (-0.08 to 0.49)		0.24 (-0.23 to 0.71)	0.20 (-0.22 to 0.61)
LoA **		-1.13 to 1.75	-1.06 to 1.70		-0.61 6o 1.13	-0.71 to 1.13		-1.74 to 2.21	-1.54 to 1.94
MAPE (%)		0.46	0.47		0.33	0.29		0.67	0.57
ICC (95% CI)		0.999 (0.995 to 1.000)	0.999 (0.996 to 1.000)		0.999 (0.997 to 1.000)	0.999 (0.998 to 1.000)		0.998 (0.994 to 0.999)	0.998 (0.995 to 0.999)
CCC		0.998	0.999		0.992	0.992		0.998	0.998

VO_2max: maximal oxygen consumption; SD: standard deviation, LoA: limits of agreement; MAPE: mean absolute percentage error; ICC: intraclass correlation coefficient; Pearson: Pearson moment correlation coefficient, CCC: Lin´s concordance correlation coefficient. MAPE and CCC were used as measures of validity, whereas ICC was used as a measure of reliability.

*ml/kg/min; **bpm
